# Sanitary Advantages of the Electric Light

**Published:** 1885-01

**Authors:** F. R. Campbell

**Affiliations:** Lecturer on Hygiene, Medical Department, Niagara University


### Sanitary Advantages of the Electric Light.

By F. R. Campbell, M. D.,

Lecturer on Hygiene, Medical Department, Niagara University.

The ventilation of public halls, churches and theatres is a matter of the greatest importance from a sanitary point of view. These places are generally used in the evening, when not only respiratory impurities, but, also, those derived from the combustion of illuminating gas vitiate the air. In devising methods of ventilation, impurities due to this latter source are seldom thought of, only the exhalations of the human body being taken into consideration. In reality, however, the products of combustion are almost, if not quite, as abundant and injurious as those of respiration. These should either be removed by the introduction of better systems of ventilation or avoided by the employment of the electric light, which neither consumes oxygen nor produces impurities.

In order to prove the advantages of the electric light in these places it will be necessary to state the evils of illumination by gas. A man exhales about six-tenths of a cubic foot of carbon dioxide in an hour. An ordinary small gas burner will give off four times that amount in the same time. To maintain the air of an enclosed space at the proper degree of purity, containing not more than six parts of carbon dioxide in 10,000, there will be required 3,000 cubic feet of air per hour for each individual and four times this amount for each gas burner, if the chandelier is not placed under a dome ventilator, a thing seldom done in churches and public halls. A church seating six hundred persons, lighted by one hundred and fifty gas burners, for example, produces as much carbon dioxide as the congregation itself. To change the air of such a place in cold weather with sufficient rapidity with any ordinary system of heating and ventilation, would be next to impossible, and the impurities will necessarily become exceedingly great, sometimes being nearly sufficient to extinguish the lights. In theatres the principal lights are usually placed in a ventilating dome, much of the impurities being thus removed. Besides, the lights burn low during the acts and the oxy-hydrogen light is now frequently used to illuminate the stage. But the cubic space allowed to each individual is usually less than in the church, which makes it all the more necessary to introduce a light producing no impurities.

Besides the carbon dioxide emitted by the gas burner, there is generally carbon monoxide, a far more injurious substance. This gas is found in considerable proportions, 7.85 per cent., in nearly all illuminating gas, and is not all converted into dioxide by combustion. Dr. Richardson says, in his recent work on preventive medicine, that this gas, diffused in small quantities from the burners in the air of badly ventilated rooms, is a frequent cause of dyspepsia, nervous disease and even diabetes mellitus. Moreover, the gas burner consumes oxygen and tends to overheat the atmosphere of a room. On this account it frequently becomes necessary in winter to restrict the amount of heated fresh air that can be admitted through the registers, and in summer the room, in a short time, becomes uncomfortably warm. The electric light, on the other hand, consumes no oxygen and produces very little heat. The danger of fire is also diminished by the use of the electric light.

Hitherto the great objections to the electric light have been its irregularity and the difficulty of controlling its intensity. These objections are valid with the Brush light, but will hardly apply to the Edison and Swan lights, where a platinum wire in a vacuum is the means of illumination. Even the Brush light is quite steady when used within doors, the great objection to its use in theatres being the inability to control its intensity. This difficulty is now overcome by using movable shades of varying translucency, so that the light may be diminished without attempting to alter the electric current. This method has been applied with success in London theatres, where the Swan light is used, a clear, manageable light being obtained with none of the injurious effects of gas. In Buffalo we have only the Brush light, but even this has been introduced into one of our theatres (Court street), thus diminishing but not avoiding the combustion of gas.

The Brush light undoubtedly produces ozone. This we have proved by actual experiment. Test papers of starch and potassium iodide were employed and the experiments conducted in a store on Main street where three Brush lights are used. The papers, when placed over the lights, rapidly became blue from the liberation of iodine by ozone. The same papers became blue, it is true, when placed outside, but not so rapidly nor so markedly. We attributed the change in the papers, when not exposed to ozone, to impurities in the potassium iodide. The production of ozone by the Edison and Swan lights is not so evident, for in them we have no electric spark passing through the air. If the test papers were not placed in the air above the light they changed no more rapidly than those outside the room. This we attribute to the fact that ozone attacks organic matter and becomes destroyed, thus purifying the atmosphere. With the recent improvements in electric lighting we may confidently expect to see the use of gas supplanted by electricity in all places of public worship and amusement.

